# Molecular Imaging of the GABAergic System in Parkinson’s Disease and Atypical Parkinsonisms

**DOI:** 10.1007/s11910-022-01245-z

**Published:** 2022-11-18

**Authors:** Miriam H. Terkelsen, Victor S. Hvingelby, Nicola Pavese

**Affiliations:** 1 Department of Clinical Medicine–Nuclear Medicine and PET Center, Aarhus University, Palle Juul-Jensens Boulevard 165, 8200 Aarhus N, Aarhus, Denmark; 2 Clinical Ageing Research Unit, Newcastle University, Newcastle Upon Tyne, UK

**Keywords:** GABA, Parkinson’s disease, Atypical parkinsonism, Neuroimaging, Positron emission tomography, Magnetic resonance spectroscopy

## Abstract

**Purpose of Review:**

During recent years, there has been a growing interest in GABAergic alterations in parkinsonian disorders. This paper aims to review the latest literature published, focusing on in vivo neuroimaging, and to suggest potential future avenues of research in the field.

**Recent Findings:**

A growing number of neuroimaging studies have focused on the association with different symptoms of Parkinson’s disease, thereby suggesting a GABAergic role in motor symptoms, gait disturbances, frontal cognition, somatic symptom disorder, and hallucinations. However, there are a number of conflicting results, and further investigations in larger, clinically well-defined cohorts are needed to elucidate possible correlations. In progressive supranuclear palsy, recent evidence suggests a decrease of GABA in the frontal lobe.

**Summary:**

In this narrative review, we discuss the possible GABAergic role in the symptoms of PD and atypical parkinsonisms and outline possible research strategies for future neuroimaging of GABAergic changes in parkinsonian disorders.

## Introduction

Idiopathic Parkinson’s disease (PD) is a neurodegenerative disease that involves multiple neuronal systems—a fact readily apparent given the broad spectrum of motor and non-motor symptoms that patients with this condition develop over time. However, the clinical diagnosis of PD is based on the classic motor symptoms bradykinesia, rigidity and resting tremor, caused by loss of dopaminergic projections from the substantia nigra (SN) to the striatum, the primary input nucleus of the motor corticostriatal circuitry. Parkinsonian symptoms are also a major feature of atypical parkinsonisms, a rather heterogeneous group of neurodegenerative diseases, that can, in the beginning, be misdiagnosed as PD. However, contrary to PD, atypical parkinsonisms, which include multiple system atrophy (MSA), progressive supranuclear palsy (PSP), corticobasal syndrome (CBS) and dementia with Lewy bodies (DLB), have a poor response to levodopa as well as additional conspicuous characteristics [1].

**Fig. 1:**
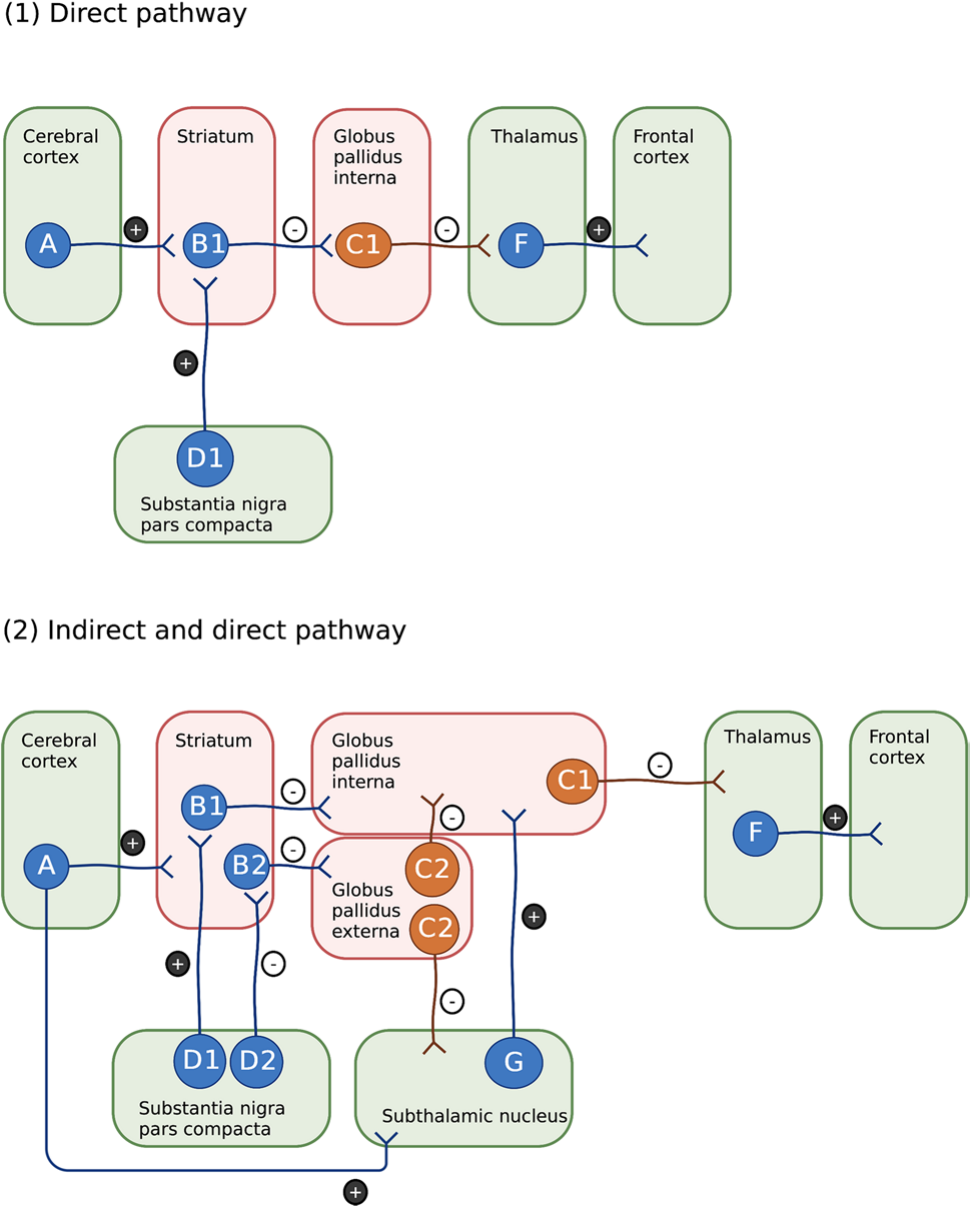
The role of GABAergic projection neurons in the basal ganglia. Striatal medium spiny neurons (B1, B2) and globus pallidal projection neurons (C1, C2) are GABAergic (marked by red areas). All blue neurons fire transiently upon excitation, while all orange neurons are tonically active. **1** In the direct pathway, transient active medium spiny neurons in putamen and caudate (B1) will inhibit the tonically active neurons of globus pallidus internal segment (C1), thereby decreasing the inhibitory outflow of the basal ganglia. The dopaminergic neurons of pars compacta substantia nigra (D1) excite striatal neurons (B1) by D1 receptors and increase the outcome of the direct pathway. **2** In the indirect pathway, transient active inhibitory, striatal neurons (B2) that project to the tonically active neurons in globus pallidus external segment (C2) have D2-dopaminergic receptors and the dopaminergic modulation from substantia nigra (D2) is therefore inhibitory. Globus pallidus external neurons (C2) project to the subthalamic nucleus and the internal segment of globus pallidus, thereby modulating the effect of the direct pathway and increasing the overall inhibitory output of the basal ganglia [2, 3] (created with Biorender.com)

Within the basal ganglia, the dopaminergic neurons from the SN pars compacta (SNc) exert modulation on the direct and indirect pathways, the two neural circuits connecting the striatum to the thalamus, decreasing the overall inhibitory output and thereby refining its processing (Fig. 1) [2, 3]. The inhibitory projection neurons of both these pathways, the projection medium spiny neurons and the efferents of globus pallidus, use γ-aminobutyric acid (GABA) as a transmitter. In fact, one-third of all brain synapses use GABA for inhibition [3].

GABA is synthesized from the excitatory neurotransmitter glutamate (via glucose, pyruvate or glutamine precursors) by the enzyme glutamic acid decarboxylase (GAD), which is only present in GABAergic neurons. At the post-synaptic membrane, GABA binds to two receptors: the ionotropic GABA_A_ and the metabotropic GABA_B_. Benzodiazepines bind to an extracellular domain on the GABA_A_ receptor to enhance GABAergic transmission. The benzodiazepine binding site on the GABA_A_ receptor is also the target for most of the established radioligands used for the *in vivo* imaging of the GABAergic system, including ^18^F-flumazenil or ^11^C-flumazenil (FMZ) and ^11^C-Ro15-4513 [4], thereby indirectly estimating the GABAergic function [3, 4].

Knowledge of GABAergic dysfunction and/or compensative regulation in PD and other neurodegenerative diseases could yield important insights into the occurrence of specific symptoms as well as disease progression and, furthermore, might bring forward new diagnostic or therapeutic options. In fact, over the years, there has been a growing interest in investigating the GABAergic system in patients with PD and atypical parkinsonisms. A recent post-mortem study found no difference in GABA_A_ receptor in the globus pallidus of brains with autopsy findings consistent with PD—but not necessarily a prior PD diagnosis—compared to controls [5], consistent with previous findings [6]. This would suggest a lack of compensatory upregulation in this part of the basal ganglia.

However, others found evidence of increased GABA_A_ receptor in the lateral part of pallidus [7]. *In vivo* imaging studies are more likely to provide information on the occurrence of changes of GABAergic function at different disease stages. Therefore, in this narrative review, we aim to give an overview of the imaging literature published on this subject and to suggest future strategies for research.

## Changes in PD

A recent study by Takashima and colleagues [8••] investigated the GABAergic and dopaminergic changes in 13 early-stage, drug-naive patients with PD compared to 15 healthy controls (HCs) using ^11^C-FMZ and ^11^C-CFT positron emission tomography (PET), the latter targeting the membrane dopamine transporter. In whole-brain analyses, they found significantly reduced binding potential of ^11^C-FMZ in the striatum as well as in frontal, parietal and temporal cortical areas, and these reductions were related to the clinical lateralization of symptoms. Therefore, the authors speculated that the reduced cortical GABAergic uptake indicates dysfunction in cortical inhibitory circuit neurons because of alterations in the nigrostriatal-cortical pathway. They furthermore reported a significant inverse correlation between putaminal GABAergic and dopaminergic radiolabelling on the more affected side in patients with PD, while this correlation in HCs was positive [8••]. This study provides evidence of a decreased striatal GABA_A_ receptor availability in PD, which possibly arises from an inhibition by local circuit neurons on dopaminergic axons and medium spiny neurons. Furthermore, it suggests an imbalance between local GABA inhibition and dopamine excitation in which the first is no longer upregulated in response to increased dopamine release. In addition, an older study by Kawabata and colleagues [9] assessed GABAergic function in PD using ^123^I-iomazenil single-photon emission computerized tomography (SPECT), which also targeted the GABA_A_ receptor. They found an inverse relationship between the availability of GABA_A_ receptors and motor symptoms in PD, especially in frontotemporal cortical regions. Degeneration of GABAergic receptors, concomitant with the general progression of the disease, would be expected to result in the findings described above. On the other hand, as the imaging studies cited above used GABA receptors as molecular targets, another possibility could be that disease progression results in a compensatory postsynaptic downregulation of GABA receptors.

During the past decade, several imaging studies using proton magnetic resonance spectroscopy (MRS) have tried to detect changes in GABAergic neurotransmission of patients with PD. MRS is a non-invasive imaging technique that measures the concentration of small molecules in the brain, and furthermore, it has the advantage that several chemical compounds can be investigated concurrently. However, using this technique for GABA detection and quantification has several limitations including poor spatial resolution due to large-size voxels and low sensitivity to synaptic changes in GABA [4]. As GABA concentration in the human brain is low, the imaging data needs to be edited to separate GABA signals from the signals of other metabolites. Most commonly used is the MEGA-PRESS editing with 3-T MR, but in general, spectral editing is limited by contamination of macromolecules. To indicate this significant contribution to the obtained signal, MRS literature often reports GABA^+^ levels [10]. In the following, however, we will not distinguish whether authors have used this term.

Reports on GABAergic alteration in the basal ganglia of patients with PD have been conflicting (Table 1). Recently, Seger and colleagues [11•] found GABA levels to be significantly elevated in the putamen of 22 patients with PD during medication ON state compared to 13 HCs. These findings are consistent with those by Emir and colleagues [12], who found GABA levels in putamen to be significantly increased with 32% in 11 patients with PD in medication OFF state compared to 11 HCs. Additionally, they found an increase of 64% in GABA levels in pons [12]. Both these studies excelled by using a 7-T MR scanner for imaging. Increased GABA levels of the basal ganglia were also found by O’Gorman Tuura and colleagues [13] in 20 patients with PD compared to 17 HCs. Furthermore, a striatal GABAergic increase is consistent with previous post-mortem findings [14] as well as MRS findings in a MPTP mouse model [15, 16].

**Table 1:** Imaging studies on GABAergic alterations in Parkinson’s disease

First author [Ref.]	Year	Imaging technique	Cohort	Disease duration, Avg., (SD), {range} in years	Medication state during imaging/clinical assessment	Results	Area of interest	GABA levels in patients with PD vs. HCs
Delli Pizzi [23]	2020	3-T MRS	42 PD/19 HC	PD: 3.5 (2.3) PD (+ SDD): 4.6 (2.1)	OFF/ON	(1) No difference between GABA levels in the medial prefrontal cortex between PD patients and healthy controls (2) GABA was elevated in patients with SDD compared to non-SDD	Medial prefrontal cortex	No difference
Dharmadhikari [20]	2015	3-T MRS	19 PD/18 HC	N/A	OFF/OFF	(1) GABA levels in the thalamus were elevated in patients, but not in the striatum	Striatum, thalamus	No difference (striatum) ↑ (thalamus)
Elmaki* [17]	2018	3-T MRS	21 PD/15 HC	3.5 (2.7)	OFF/OFF	(1) GABA levels in the basal ganglia were lower in patients than in controls	Basal ganglia	↓
Emir [12]	2012	7-T MRS	13 PD/12 HC	N/A	OFF/OFF	(1) GABA levels in the pons and putamen were higher in patients	Pons and putamen	↑
Firbank [30]	2018	3-T MRS	36 PD/20 HC	No hallucinations: 9.6 (6.5) With hallucinations: 11.0 (7.4)	N/A	(1) Higher GABA levels in patients with PD and hallucinations compared to PD patients without hallucinations and healthy controls	Visual cortex	N/A
Gong* [18]	2018	3-T MRS	22 PD/16 HC	PIGD: 3.3 (1.7) TD: 3.8 (3.4)	N/A	(1) GABA levels were lower in patients with PD (2) Inverse correlation in PIGD patients between GABA levels and UPDRS	Basal ganglia	↓
Gröger [24]	2014	3-T MRS	21 PD/24 HC	[3–7, 8••, 9, 10, 11•, 12, 13]	N/A	(1) Slight, non-significant elevation of GABA in substantia nigra	Substantia nigra	No difference
Kawabata [9]	1996	^123^I-iomazenil SPECT	15 PD	N/A	N/A	(1) Inverse relationship between the availability of GABA_A_ receptors and motor symptoms	Cortex	N/A
van Nuland [22•]	2020	3-T MRS	60 PD/22 HC	N/A	ON and OFF/ON and OFF	(1) No alterations in GABA levels due to PD, clinical phenotype with tremor or medication	Thalamus, motor cortex and visual cortex	No difference
O’Gorman Tuura [13]	2018	3-T MRS	20 PD/17 HC	9.25 (4.3) [2–7, 8••, 9, 10, 11•, 12–18]	ON/ON	(1) Elevated GABA levels in the basal ganglia of patients (2) This elevation in GABA correlated with the degree of gait disturbance (3) No difference in prefrontal GABA levels between groups	Basal ganglia	↑
Oz [25]	2006	4-T MRS	10 PD/11 HC	2.3 (1.5)	OFF/ON	(1) No difference between GABA levels in patients compared to healthy controls	Substantia nigra	No difference
Pesch [19]	2019	3-T MRS	35 PD/35 HC	4.7	ON/ON	(1) No difference between GABA levels in patients compared to healthy controls	Basal ganglia, thalamus	No difference
Piras [29]	2020	3-T MRS	20 PD/20 HC	3.51 (1.78)	ON/ON	(1) In the right cerebellar hemisphere, there was an inverse correlation between GABA levels and cognitive scoring on the Stroop word-color test in patients with PD, while this was positive in healthy controls (2) This correlation was positive in both groups in analyses of the left cerebellar hemisphere and the overall mean of cerebellum	Cerebellum	No difference
Takashima [8••]	2022	^11^C-FMZ PET	13 PD/15 HC	13.8 (8.1)	OFF/OFF	(1) GABAergic binding potential was reduced in the striatum as well as frontal, parietal and temporal cortical areas of patients (2) There was an inverse correlation between GABAergic and dopaminergic signals in the putamen in patients	Striatum and frontal cortex	↓
Trujillo [37]	2022	3-T MRS	14 PD/19 PD + ICB	PD: 7.2 (4.2) PD + ICB: 4.3 (3.3)	OFF and ON/OFF and ON	(1) Patients with ICB had a reduced GABAergic response to dopaminergic therapy only in the thalamus	Thalamus, motor cortex	N/A
Seger [11•]	2021	7-T MRS	19 PD/13 HC	5.7 (4.2)	ON/OFF and ON	(1) GABA levels in putamen were significantly elevated in patients (2) Patients’s putaminal GABA correlated inversely with dopaminergic treatment response	Putamen	↑
Song** [26]	2021	3-T MRS	18 PD/18 HC	2.9 (1.5)	OFF/ON	(1) GABA levels in the upper brainstem were lower in patients than in controls	Upper brainstem	↓
Song** [32]	2021	3-T MRS	11 PD/11 HC	3.4 (1.8)	OFF and ON/OFF and ON	(1) GABA levels were lower in patients than controls (2) Dopaminergic therapy improved GABA levels in the upper brainstem of patients	Upper brainstem	↓

Twelve out of 17 papers found in our literature search were published within the last 5 years. Upwards arrow (↑) indicates significant or non-significant elevation of GABA in patients with PD compared to HCs. Downwards arrow (↓) indicates a significant decrease of GABA level in patients with PD compared to HCs

*T* tesla, *MRS* magnetic resonance spectroscopy, *PD* Parkinson’s disease, *HCs* healthy controls, *N/A* information not available, *PET* Positron emission tomography, ^*11*^*C-FMZ* ^11^C-flumazenil, *SDD* somatic symptom disorder, *ICB* impulsive compulsive behaviour

^*^^/^**Possible overlap in the participants

On the other hand, Elmaki and colleagues [17] found levels of GABA in the left basal ganglia of 21 patients with PD in medication OFF state to be significantly lower compared to 15 HCs. The authors suggested that rather than GABAergic neuronal cell loss, this may be a compensatory inhibition of GABAergic interneurons as response to nigral dopaminergic cell loss and striatal dopaminergic receptor upregulation. While the authors did report lower GABA levels in PD patients, the reported confidence intervals for cases and controls were overlapping. Another report by co-authors of the study, Gong and colleagues [18], supported decreased basal ganglia GABA levels in 22 patients with PD compared with 16 HCs. Unfortunately, it is not clear whether there could be an overlap in the cohorts of these two reports.

Pesch and colleagues [19] investigated GABA in the basal ganglia and the thalamus with MRS in an all-male cohort of 35 patients with PD and 35 HCs. They did not find evidence of any difference in GABA levels in either region of patients compared to HCs. Conversely, Dharmadhikari and colleagues [20] found significantly elevated GABA levels in the thalamus of 19 patients with PD compared to 18 HCs. However, no difference was found in the striatum. Furthermore, they found a significant positive correlation between thalamic GABA levels and Unified Parkinson’s Disease Rating Scale (UPDRS) scores which suggest that increased output from the internal segment of globus pallidus to the thalamus plays a role in PD motor symptoms [21].

In a larger cohort, van Nuland and colleagues [22•] investigated GABA in the thalamus, motor cortex and visual cortex of 60 PD patients and 22 HCs. They found no significant differences between patients and controls. Delli Pizzi and colleagues [23] did not find any difference in GABA levels in the medial prefrontal cortex in 19 patients compared to 19 HCs. Gröger and colleagues [24] found substantia nigra GABAergic levels to be slightly, however not significantly, elevated in 21 patients with PD compared to 24 HCs, while Oz and colleagues [25] found no difference between substantia nigra GABA levels in 10 patients with PD compared to 11 HCs.

Song and colleagues [26] targeted GABA in the upper brainstem of 18 patients with PD compared to 18 HCs. They found patients to have significantly reduced levels of GABA in the brainstem, and further demonstrated that there were no significant differences in metabolites of other investigated transmitters. This suggests overall conservation of tissue, and thus, the reduced GABA levels could be due to decreased production and not neuronal loss. A decreased GABAergic signal in the brainstem is possible in opposition to the previously mentioned significant elevation found by Emir and colleagues [12] in pons. Their methods differed to some extent, including that Emir and colleagues [12] used linear combination modelling (opposed to J-difference editing) to quantify GABA, a smaller region of interest, and that they targeted pons, which is located lower in the brainstem. Concerning non-motor symptoms of PD, assessed by the Non-Motor Symptom Questionnaire [27], Song and colleagues [26] found no correlation with GABA brainstem levels.

## GABA in Relation to PD Symptoms

Patients with PD have a large variability in symptoms and disease progression. Several studies have investigated the role of GABAergic changes in different clinical symptoms.

### Tremor

Van Nuland and colleagues [22•] divided PD patients into subgroups with dopamine-resistant tremor (*n* = 17), dopamine-responsive tremor (*n* = 23) or no tremor (*n* = 20). They found a significant, inverse correlation between motor cortex GABA and Movement Disorder Society Unified Parkinson’s Disease Rating Scale (MDS-UPDRS) [28] motor scores, which suggests that motor symptom severity is related to GABA concentrations in the motor cortex. Only in tremor-dominant patients, they found a significant negative correlation between thalamic GABA levels and disease severity. Thus, the pathophysiology of PD might be modulated by GABA, for instance by enhanced excitability in the primary motor cortex interfering with normal processing, and furthermore, may be independent of dopaminergic medication.

### Impaired Cognition and Hallucinations

During disease progression, most patients with PD will experience cognitive impairments, ranging from mild cognitive impairment (MCI) to dementia. In a recent PET imaging study, Takashima and colleagues [8••] found a positive correlation between GABAergic function in the frontal cortex and executive function (assessed by the Frontal Assessment Battery score) in early-stage PD, but not with the global cognition score Mini-Mental State Examination which was normal in this patient cohort. In the cerebellum of 20 patients with PD compared to 20 HCs, Piras and colleagues [29] found that right cerebellar hemispheric GABA levels, assessed with MRS, were negatively correlated with performance on the Stroop word-color test and, conversely, in HCs. For the left hemisphere and the cerebellum overall mean, both PD patients and HCs showed a positive correlation [29].

Firbank and colleagues [30] used MRS to describe occipital GABA levels in 17 patients with PD compared to 19 patients with PD and hallucinations and found significantly higher GABA in the latter. This difference remained significant after correction for cognitive score even though patients with hallucinations had worse cognition and were more likely to take cholinesterase inhibitors. All patients had impaired cognition, MCI (*n* = 15) and PD dementia (*n* = 21). Furthermore, they found evidence of grey matter loss in the anterior temporal lobe as well as in the V4 region of the visual cortex on MRI. The authors suggest that decreased levels of inhibitory GABA are adaptive to poor visual input to maintain recognition of objects at the cost of visual hallucinations. Decreased GABA levels in the primary visual cortex have previously been found post-mortem in patients with DLB [31].

### Somatic Symptom Disorder

Delli Pizzi and colleagues [23] used MRS to investigate the pathophysiology of the somatic symptom disorder (SDD), which is more prevalent in patients with PD than in the general population. In a cohort of 19 patients with PD, 23 patients with PD and SDD, 19 HCs and 14 patients suffering only from SDD, they found participants with SDD (*n* = 37) to have significantly higher GABAergic signals in the medial prefrontal cortex compared to participants without (HCs or patients with PD only). This suggested that alterations in the prefrontal GABAergic system play a role in the production of dysfunctional integration of body perception.

### Axial Symptoms

The axial symptoms of PD are motor symptoms beyond the classical triad, where dopaminergic treatment is often ineffective. These include gait problems, such as freezing of gait, and problems with balance, posture, speech and swallowing. O’Gorman Tuura and colleagues [13] examined whether GABAergic alterations in the basal ganglia and prefrontal cortex could be correlated to axial symptoms in a cohort of 20 patients with PD and 17 HCs. The severity of axial symptoms was determined during medication ON state using a MDS-UPDRS gait subscore, containing both subjective and objective items. MRS scans were made when patients were due to take their next dose of dopaminergic medicine; however, no pause was made. GABA levels in patient’s basal ganglia were positively correlated to the gait subscore as well as to the MDS-UPDRS gait item (3.10). When they divided patients into subgroups of akinetic-rigid and tremor-dominant phenotypes, both correlations were present at trend level only in the akinetic-rigid subgroup. These correlations, however, did not remain significant when adjusted for the levodopa equivalent dose. Positive correlations were also found in the akinetic-rigid subgroup between basal ganglia GABA levels and the MDS-UPDRS item concerning difficulties arising from a chair as well as the item concerning posture, respectively, and these correlations remained significant after adjustments. The authors speculated that the elevated GABA levels in the basal ganglia could be indicative of an excessive inhibition of processing of sensory inputs which are crucial in normal posture and locomotion, thereby contributing to gait disturbances. They argued that this theory of a GABAergic role in gait problems could be further investigated by examination of the midbrain locomotor region. Emir and colleagues [12] did find elevated GABA levels in pons; however, they did not report any investigation of a possible relation to axial symptoms.

On the contrary, Gong and colleagues [18] found significantly higher basal ganglia GABA levels in a subgroup of patients with postural instability gait difficulty (PIGD, *n* = 13) compared to tremor-dominant (TD, *n* = 9) patients. They found a significant inverse correlation in PIGD patients between UPDRS and GABA levels. The conflicting reports by O’Gorman Tuura et al. [13] and Gong et al. [18] indicate that more knowledge on the PD phenotypic subtypes is needed, preferable in larger, very well-characterized cohorts.

## Response to Levodopa Treatment

In a cohort of 11 patients with PD and 11 HCs, Song and colleagues [32] investigated the effect of dopaminergic therapy on the GABAergic system in the upper brainstem. MRS and UPDRS scoring in patients were performed while medicine depleted and after administration of 150–200% effective morning dose with up to 2 h between scans. Controls were only examined once. Upper brainstem GABA levels were significantly lower in patients than in controls, and they had a significant increase in GABA towards normalization after administration of levodopa. As anticipated, levodopa treatment also gave a significant improvement in motor symptoms. However, this improvement was not correlated to change in GABA. Others have previously found that levodopa administration stimulated GABA release in HCs using PET imaging [33]. Song and colleagues [32] speculate that motor symptoms in PD are caused by increased activity in the basal ganglia due to downregulation of GABAergic nigrostriatal projection neurons. However, the downregulation of GABAergic inhibition in the upper brainstem could also be a response to the diminished dopaminergic increase and decrease of the basal ganglia direct and indirect pathways, respectively.

In the striatum, GABA modulates release from dopaminergic neurons by axonal GABA receptors and the dopaminergic terminals may even co-release GABA to modulate their dopaminergic effect [34–36]. Seger and colleagues [11•] performed a clinical analysis of treatment response with MDS-UPDRS motor score in their cohort of patients with PD (*n* = 22) in ON and OFF states, the latter obtained by medication withdrawal for at least 14 h. In the putamen, they found that GABA levels correlated negatively with the dopaminergic treatment response, thereby indicating a possible mechanism of medical treatment refraction [11•].

In their cohort of 60 patients with PD, van Nuland and colleagues [22•] examined the GABAergic system in the thalamus, motor cortex and visual cortex. They performed clinical assessments with MDS-UPDRS and MRS scans during both dopaminergic medicine depletion and after administration of an average medication dose of 170% normal morning dose. They did not find any effect of levodopa on GABA levels in the targeted areas [22•].

Trujillo and colleagues [37] investigated a cohort of patients with PD in both medication ON and OFF states, 19 with and 14 without impulsive compulsive behaviour (ICB). They found that only the patients with ICB had a significantly reduced GABAergic response in the thalamus to dopaminergic treatment, and that the self-reported rating of impulsivity correlated inversely to the thalamic GABAergic changes [37].

## Changes in Atypical Parkinsonism

The imaging research into GABAergic system changes in the atypical parkinsonisms is focused on PSP (Table 2). Recently, Adams and colleagues [38••] hypothesized that accumulating tauopathy results in GABAergic cell loss, like the cell degeneration seen in the behavioural variant of frontotemporal dementia (FTD), giving rise to the distinctive clinicopathological phenotypes of these diseases. To test this hypothesis, the authors used MRS to compare frontal GABA levels in patients with FTD (*n* = 17) and PSP (*n* = 15) with 30 HCs, noting a significant decrease in GABA concentration in PSP patients. Participants in the study were further subjected to an assessment of dynamic causal modelling of magnetoencephalography (MEG) to identify local network dynamics, and the functional implication of the relative GABA deficiency was assessed. While a comprehensive exposition of this methodology is beyond the scope of this review, the main findings will be summarized here. Overall, the authors found deficient frontotemporal connectivity in patients compared to controls. They further report that the extent of the extrinsic connectivity aberration scaled with cognitive disability. The intrinsic connectivity of the frontal cortical regions (and by implication, the intrinsic GABAergic microcircuits) was similarly aberrant in the two patient groups. Interestingly, these changes were differentially affected by an increase in GABAergic transmission by the GABA-reuptake inhibitor tiagabine in PSP compared to FTD. These findings imply that GABAergic transmission loss might play a pathophysiological role in PSP, although a possible causality of such a relationship remains to be elucidated. While the study by Adams and colleagues [38••] seems to imply an instrumental role for GABA transmission in the development and symptomatology of PSP, a similar MRS study performed by Bonnet and colleagues [39], with seven patients with PSP compared to eight HCs, found no statistically significant differences in GABA levels.

**Table 2:** Imaging studies on GABAergic alterations in atypical parkinsonisms

First author [Ref.]	Year	Imaging technique	Cohort	Results	Area of interest	GABA levels in patients with PSP vs. HCs
Adams [38••]	2021	7-T MRS	11 PSP/20 HC	(1) Frontal GABA levels were decreased in patients with PSP	Frontal cortex	↓
Foster [40]	2000	^11^C-FMZ PET	12 PSP/10 HC	(1) Global GABAergic binding potential was reduced at 13% of patients (2) The greatest reduction of 20% was seen in the anterior cingulate cortex	Anterior cingulate gyrus	↓

Downwards arrow (↓) indicates a significant decrease of GABA level in patients with PD compared to HCs

*T* tesla, *MRS* magnetic resonance spectroscopy, PSP progressive supranuclear palsy, *HCs* healthy controls, *PET* Positron emission tomography, ^*11*^*C-FMZ* ^11^C-flumazenil

In a PET imaging study, Foster and colleagues [40] reported absolute global ^11^C-FMZ binding to be significantly reduced at 13% in 12 patients with PSP compared to 10 HCs. In area analyses, the reduction was greatest in the anterior cingulate cortex of the patients with PSP, a region in which ^18^F-FDG also showed relative frontal hypometabolism in these patients. The authors suggested loss and deafferentation of cortical neurons containing GABA_A_ receptors to be contributing to these metabolic changes [40].

## Future Research

The research field of the GABAergic changes in parkinsonian diseases still has many unanswered questions, and there is no doubt *in vivo* neuroimaging has an important role to play. Variations among results of parkinsonian GABAergic alterations might be due to a variation in imaging acquisition and regions targeted, as well as various objectives and variation in clinical phenotypes. Most papers, discussed here, had small numbers of participants, and future studies are needed to confirm their findings. Preferably, future research should aim to describe GABAergic alterations in relation to other pathological changes such as correlation with symptoms, relation to dysfunction in transmission of other neurotransmitters, changes in GABA transmission over time, or interaction with treatment regimens.

The GABAergic neurons of the basal ganglia inhibit their target neurons in different manners, and it would therefore be prudent to be able to distinguish changes isolated to these anatomical structures. This is a great challenge in MRS imaging as the volume of interest is too large to distinguish, e.g. the anterior ventrolateral nucleus from the posterior ventrolateral nucleus in the thalamus or even a striatal signal from a pallidal neuron. Keeping in mind the signalling pathways of the basal ganglia, an upregulated release of striatal, inhibitory interneurons could diminish the transient signal of medium spiny neurons towards tonically inhibitive pallidal neurons or the striatal dopaminergic release [34], thereby increasing the overall basal ganglia inhibitory output. On the contrary, an increased GABAergic signal in the internal globus pallidus could represent upregulation of striatal medium spiny neurons and a decrease in basal ganglia output. Our current understanding of dopaminergic and GABAergic interactions in the basal ganglia is probably lacking [2]. High-resolution PET imaging might be able to give a more detailed insight into the parkinsonian alterations of these complex neuronal networks as tracer binding gives a more specific evaluation of synaptic GABA [4].

It also remains to be established whether changes in GABA are part of the widespread neurodegenerative process in PD, the result of compensatory adjustments to the neurodegeneration of other neurotransmitter systems or a combination of both processes. In fact, an intrastriatal downregulation of local GABAergic interneurons could be the first response to decreased dopaminergic modulation. Takashima and colleagues [8••] reported a putaminal GABAergic decrease in a cohort of patients with PD, who, for the most part, were at Hoehn and Yahr (HY) stage 1 despite a long disease duration (Table 1) [8••]. Studies reporting a striatal increase in GABA only included patients with HY stage 2 [11•, 12]. Future research should, therefore, include longitudinal investigations of striatal GABAergic alterations during disease progression.

Recognizing that multiple neuronal systems and their interaction are affected in neurodegenerative diseases, future research should target multiple neurotransmitters in attempt to further describe these contingencies of disease progression. Neuroimaging offers the opportunity to investigate the interaction of neurotransmitter molecules [8••, 24]. A disadvantage to PET imaging is the lack of a tracer that directly targets the GABA molecule. To date, tracers only target GABA indirectly by presynaptic vesicles or postsynaptic receptors; however, the combination of both presynaptic and postsynaptic targets ought to be of interest.

As clinical phenotype and progression of symptoms differ greatly among patients with PD, it is important to characterize both motor and non-motor symptoms alongside neuroimaging. GABAergic dysfunction of the basal ganglia has been implicated in axial dysfunction [13], however, only in a small, exploratory subgroup analysis. To divide patients with PD into phenotypical subgroups, larger cohorts are needed. Furthermore, when comparing symptoms to imaging, the state of ON or OFF medication should be considered. Clinical assessments are often made on medication while the imaging is made while patients are medicine depleted. As such, more information on how dopamine replacement affects the GABAergic systems is needed.

An important avenue of research, not clearly pursued in any of the reviewed literature, is the relation of changes in GABAergic transmission and its causal relation to overall disease progression. As evidenced by the studies included, reported changes are of a varied and heterogeneous nature. Future research should seek to identify and explain the extent to which aberrant GABAergic transmission is an etiological contributor to the development of PD and related disorders and, contrarily, the extent to which any alterations over time represent a compensatory mechanism to the disease progression. If the latter is the case, it remains to be seen whether clinical phenotypes bear any relation to these compensatory changes being of a beneficial or dysregulated and detrimental nature.

Impaired sleep is very common in patients with PD, including insomnia, excessive daytime sleepiness with sleep attacks, restless legs syndrome and rapid eye movement sleep behaviour disorder (RBD). While dopaminergic medicine may relieve some of these symptoms, some patients experience worsening especially due to higher doses or during increasing dose regimens of dopaminergic agonists, where the risk of sudden sleep attacks makes driving prohibited [41]. Increased levels of GABAergic signal in the medial prefrontal cortex have been implicated to play a role in narcolepsy [42], and benzodiazepines targeting the GABA_A_ receptor are used in the treatment of acute insomnia. Surprisingly, to the best of our knowledge, there have been no imaging studies on GABAergic alterations in sleep disturbances in Parkinson’s disease. Isolated RBD is considered a very important prodromal marker of parkinsonian diseases, as a great number of patients with RBD will progress to a neurodegenerative disorder, primarily PD and DLB, and less frequently MSA [43]. One of the treatment options for RBD is the benzodiazepine clonazepam, and evidence from research in animals suggests GABA_A_ receptor and glycine inhibition to trigger RBD symptoms [44, 45].

## Conclusion

It has not yet been established whether changes in GABA are part of the widespread neurodegenerative process in PD and atypical parkinsonisms or if they are a compensatory consequence of the dopaminergic degeneration, as suggested by several of the studies presented here. PET imaging studies targeting GABA are limited in both PD and atypical parkinsonisms. The field needs studies with larger cohorts with clinically well-defined phenotypes and, furthermore, the use of high-resolution PET imaging to detect variation in changes between different structures of the brain.
